# A Wideband Circularly Polarized Dipole Antenna with Compact Size and Low-Pass Filtering Response

**DOI:** 10.3390/s24123914

**Published:** 2024-06-17

**Authors:** Xianjing Lin, Zhangrun Weng, Yibin Hong, Yao Zhang

**Affiliations:** 1 School of Electronic Engineering and Intelligence, Dongguan University of Technology, Dongguan 523808, China; linxj@dgut.edu.cn (X.L.); 15918909380@163.com (Z.W.); yibin_hong@163.com (Y.H.); 2 Institute of Electromagnetics and Acoustics, Xiamen University, Xiamen 361005, China

**Keywords:** wideband antenna, cross-dipole antenna, circularly polarized antenna, filtering antenna

## Abstract

This paper presents a compact wideband circularly polarized cross-dipole antenna with a low-pass filter response. It consists of two pairs of folded cross-dipole arms printed separately on both sides of the top substrate, and the two dipole arms on the same surface are connected by an annular phase-shifting delay line to generate circular polarization. A bent metal square ring and four small metal square rings around the cross-dipoles are employed to introduce new resonant frequencies, effectively extending the impedance and axial-ratio bandwidth. Four square patches printed on the middle substrate are connected to the ground plane by the vertical metal plates in order to reduce the antenna height. Thus, a compact wideband circularly polarized antenna is realized. In addition, a transmission zero can be introduced at the upper frequency stopband by the bent metal square rings, without using extra filter circuits. For verification, the proposed model is implemented and tested. The overall size of the model is 90mm×90mm×33mm ($0.37\lambda_{0}\times 0.37\lambda_{0}\times 0.14\lambda_{0}$; $\lambda_{0}$ denotes the center operating frequency). The measured impedance bandwidth and 3 dB axial-ratio (AR) bandwidth are 53.3% and 41%, respectively. An upper-band radiation suppression level greater than 15 dB is realized, indicating a good low-pass filter response.

## 1. Introduction

Compared with linearly polarized antennas, circularly polarized antennas can receive arbitrarily polarized incoming waves, and, therefore, they are widely used in wireless communication, radar, radio frequency identification and satellite communication systems [1].

Cross-dipole antennas have been widely studied for their ability to achieve good circular polarization [2,3,4,5,6,7,8,9,10,11,12,13,14,15,16,17,18,19,20,21,22,23,24]. The traditional cross-dipole circular polarization antenna is connected by two annular delay-line phase shifters to the four narrow arms of the cross-dipole and fed through the coaxial line, which can achieve a 3 dB axial-ratio bandwidth of about 7% [2,3]. In order to broaden the axial-ratio bandwidth, the cross-dipole arms can be changed into wide rectangular [4], wide ring [5], bowtie [6] and the divided wide dipole arms [7]. Or, using the folded microstrip lines instead of annular delay line phase shifters [8], or parasitic units [9,10,11] around the dipole arms, the axial-ratio bandwidth can be expanded to 25–90%. However, for these designs, the wide axial-ratio bandwidth is usually obtained through increasing antenna size or height, or unstable radiation patterns. In order to reduce the antenna size, the most direct way is to fold the cross-dipole arms [12,13,14,15,16,17,18] or the feed network [19], but this results in limited axial-ratio bandwidth (7–13%). Using coupled slips instead of annular delay-line phase shifters and loading stepped square rings can effectively reduce the plane size and maintain a wide axial-ratio bandwidth [20], but the antenna height is stable at 0.25 $\lambda_{0}$. The antenna height can be reduced from 0.25 $\lambda_{0}$ to around 0.16 $\lambda_{0}$ by loading a short-circuited wall at the radiation patch [21] or using SIW feed network [22] and AMC reflectors [23,24]. However, these methods can result in large plane sizes or high cross-polarized radiation, which leads to the radiation pattern deterioration. For cross-dipole circularly polarized antennas, it is still challenging to realize a compact structure while maintaining a wide axial-ratio bandwidth and stable in-band radiation pattern.

In this work, by folding narrow cross-dipole arms and introducing a bent square ring and four small metal square rings around the dipole arms, the plane size of the antenna can be effectively reduced and 41% of the axial-ratio bandwidth can be obtained. Then, the profile height can be reduced to 0.14 $\lambda_{0}$ by introducing four patches connected by the metal plates around its outer right-angle edges to the ground plane. In addition, the bending metal square ring can not only widen the axial-ratio bandwidth but also introduce a radiation zero at the high-frequency stopband, achieving a good low-pass filtering response.

## 2. Antenna Structure and Design Procedure

### 2.1. Antenna Structure

**Figure 1:**
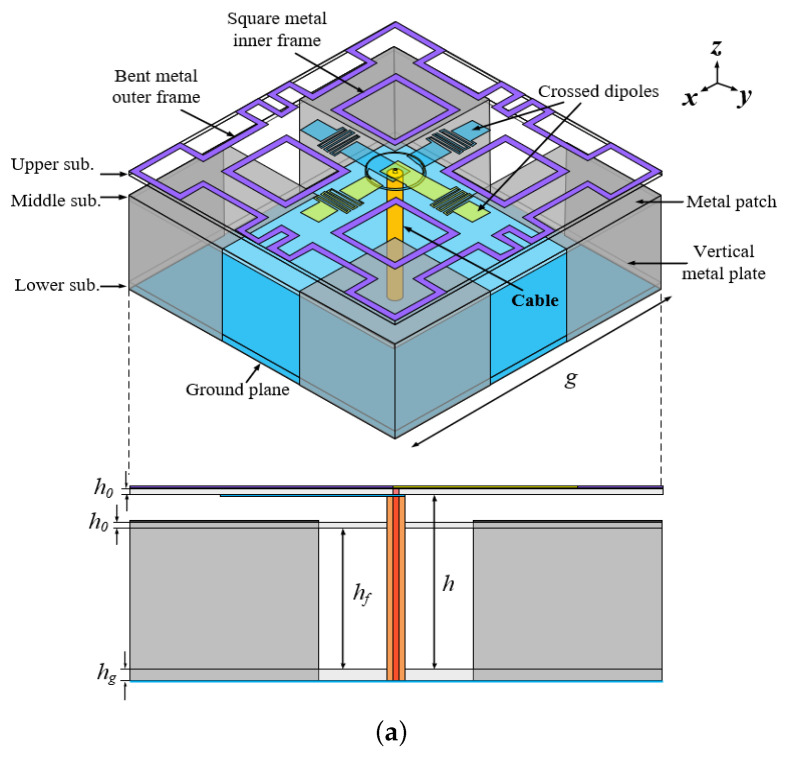

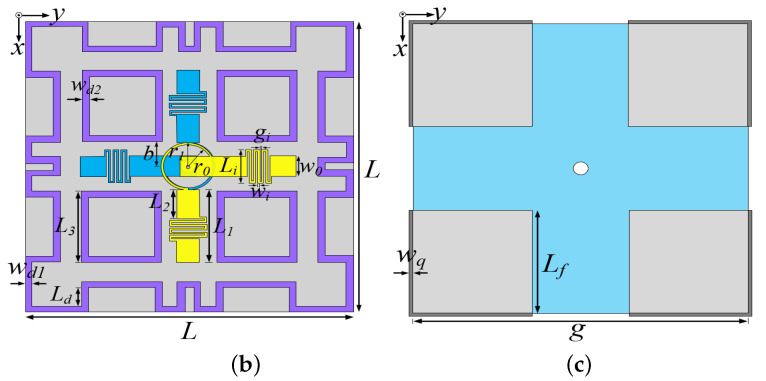
Antenna structure, (**a**) total view, (**b**) dipole antenna layer top side and (**c**) middle dielectric layer top side. ($g=90,L=90,L_{1}=22,L_{2}=16,L_{3}=22,L_{f}=32,w_{0}=6,L_{i}=10,w_{i}=0.5$, $g_{i}=0.6,r_{0}=6.9,r_{1}=7.1,b=7.5,w_{d1}=1.8,w_{d2}=2,L_{d}=6,h=30,h_{f}=25,h_{0}=1$, $h_{g}=2$, all in mm).

The structure of the proposed compact wideband circularly polarized cross-dipole antenna with low-pass filter response is shown in Figure 1. It consists of two folded cross-dipoles, a bent metal outer square ring, four metal inner square rings, four square metal patches and four vertically grounded metal plates. The antenna is fed by coaxial cables. The folded cross-dipole arms are printed separately on both sides of the top substrate, and a bent metal outer square ring and four-square metal inner square rings are printed around the dipole arms on the upper surface. Four square patches are printed on the upper surface of the middle substrate, and four vertical metal plates are connected at the outer right angles of the four-square patches. A metal ground plane is printed on the lower surface of the bottom substrate, the coaxial inner conductor is connected to the dipole arm printed on the upper surface of the top substrate, and the outer conductor is connected to the dipole arm printed on the back side. The FR4 epoxy substrate ($\varepsilon_{r}=4.4$, tanδ = 0.02) of height 1 mm is used for the design.

### 2.2. Design Procedure

**Figure 2:**
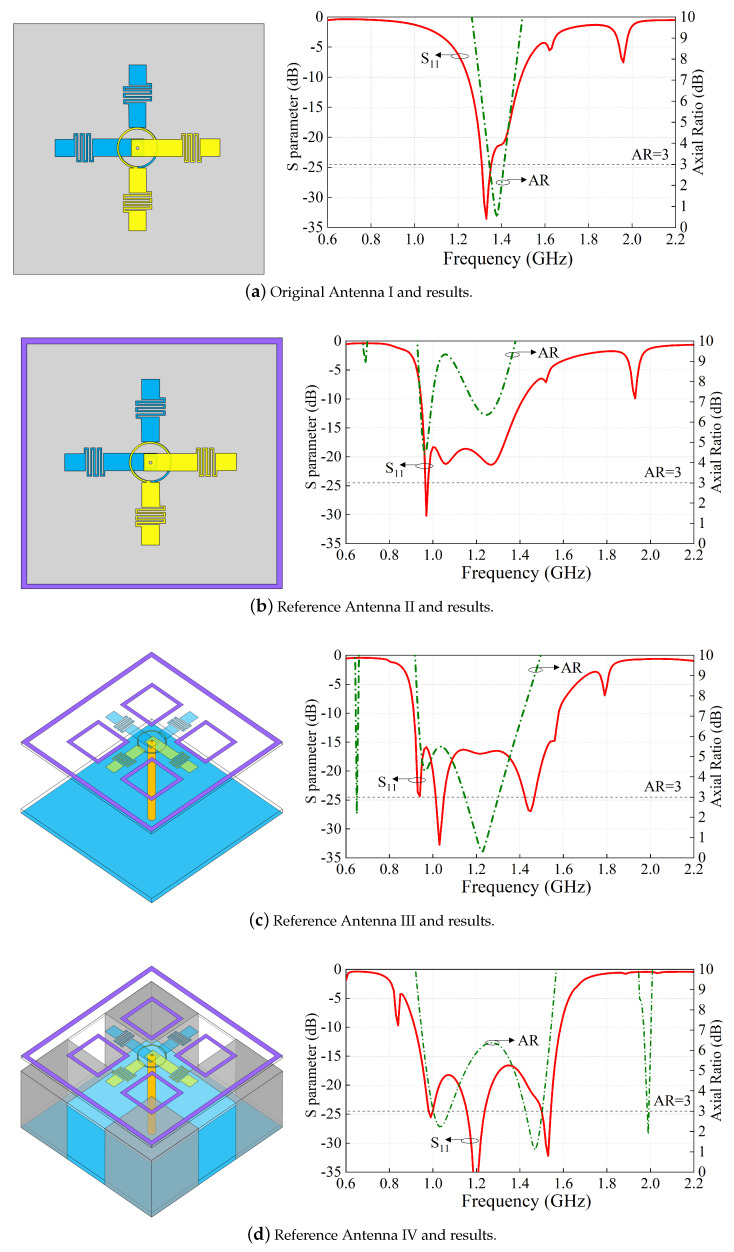

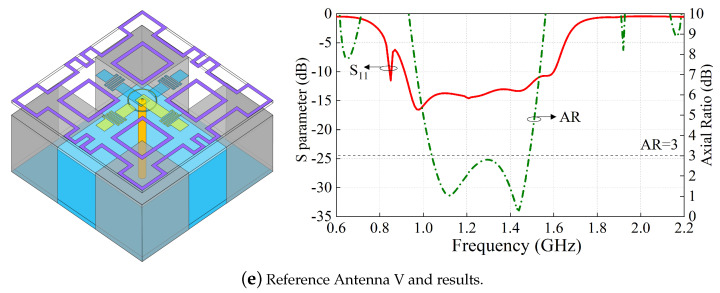
Structure and results of five antennas.

**Figure 3:**
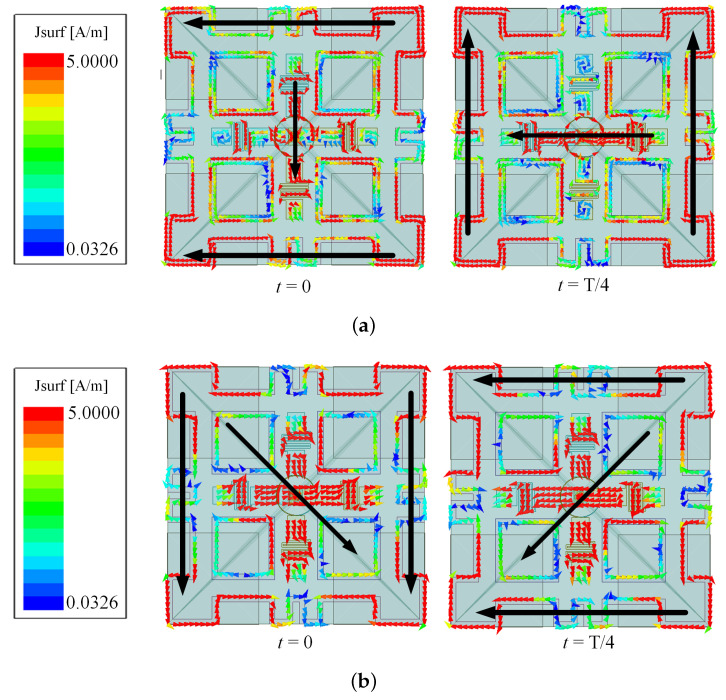

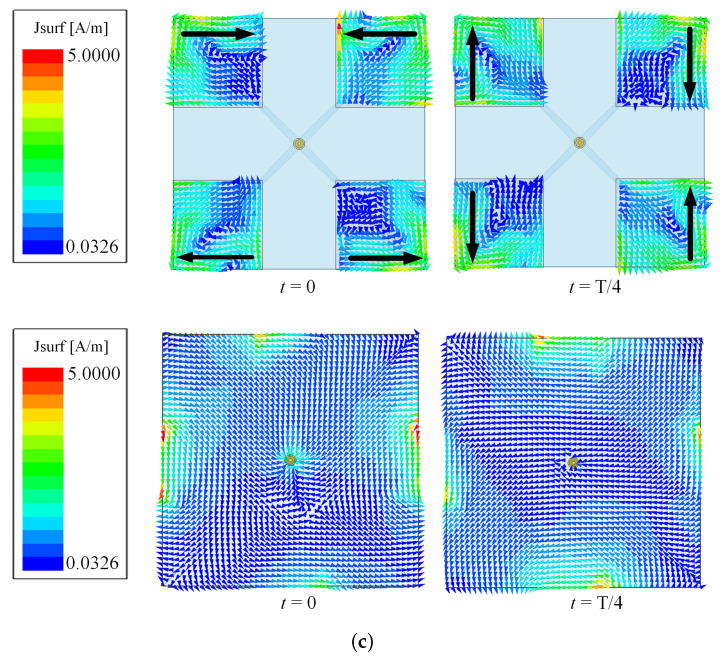
Surface current distribution of the reference Antenna V when the phase is 0° and 90° at operating frequency (**a**) 1.12 GHz, (**b**) 1.44 GHz and (**c**) 1.3 GHz.

To reveal the working principle of the proposed antenna, Figure 2 shows five reference antennas and corresponding S-parameter and axial ratio results. As seen in Figure 2a, it is a folded cross-dipole arm CP antenna structure. The S-parameter and AR values are also illustrated in Figure 2a. It can be seen that the 10 dB impedance band ranges from 1.25 to 1.49 GHz (17.5%), with only one CP resonant frequency in the passband, and the 3 dB axial-ratio bandwidth (5.1%) is very limited. When a large square ring is loaded at the periphery of the cross-dipole arms, the overall operation frequency band is shifted to the lower band, and the impedance bandwidth is extended to 39.66% (0.95–1.42 GHz). Meanwhile, two CP resonant frequencies in the passband have emerged, but the circular polarization performances are not good, as seen in Figure 2b. To improve the circular polarization performance, four metal square rings are introduced between the large square ring and the cross-dipole, as observed in Figure 2c. In this case, the plane size and antenna height are very large. Then, the reference Antennas IV and V have been developed. By adding a middle substrate right under the cross-dipole and printing four square patches at the upper surface of the substrate, then connecting the patches to four vertically grounded metal plates, the profile height of the antenna can be effectively reduced with the performance nearly unchanged. Figure 2d shows the structure of the reference Antenna IV and its results. To further reduce the plane size of the antenna, the large square ring is bent, and the performance is still unchanged, as illustrated in Figure 2e. Hereto, the compact wideband CP cross-dipole antenna is realized. Figure 3a,b show the current distributions of the radiator at the two CP resonant frequencies of 1.12 GHz and 1.44 GHz, respectively. And Figure 3c shows the current distributions of the four small metal square rings and the ground plane at the center frequency of 1.3 GHz when phase = 0° and 90°. As observed, the antenna operates as an LHCP antenna. It should be mentioned that the current of the four square patches printed on the middle substrate is canceled, and the coupling current to the ground by the four vertically grounded metal plates is little. Thus, the ground current has little effect on the radiation of the antenna.

### 2.3. Low-Pass Filtering Radiation Response

**Figure 4:**
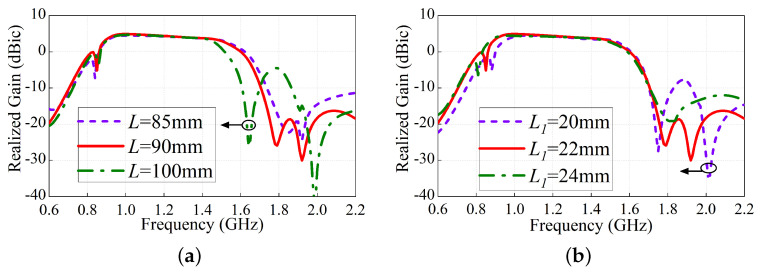
Frequency control of the radiation nulls at (**a**) 1.78 GHz and (**b**) 1.92 GHz.

The antenna-realized gain against the ring parameters *L* and $L_{1}$ is illustrated in Figure 4. Low-pass type radiation response is seen with two controllable upper-band radiation nulls. As presented in Figure 4a, the upper-band radiation $Null_{1}$ frequency increases from about 1.62 GHz to 1.82 GHz when *L* (the length of the bent square ring) deceases from 100–85 mm, while the upper-band radiation $Null_{2}$ frequency is nearly fixed. Similar phenomenon can be seen when the folded cross-dipole arm $L_{1}$ changes. Both of the two nulls are introduced at the upper-band, which makes the proposed antenna have a good low-pass filtering response.

**Figure 5:**
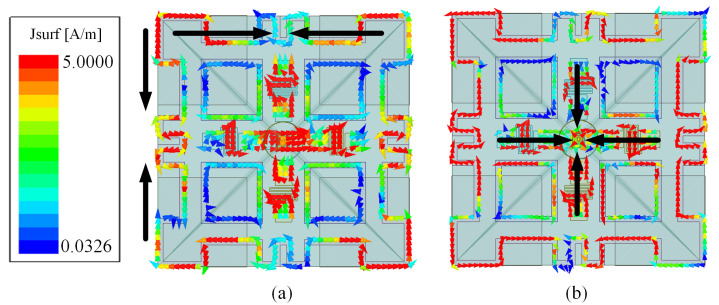
Current distributions on the reference Antenna V at the two null frequencies (**a**) $Null_{1}$ 1.78 GHz and (**b**) $Null_{2}$ 1.92 GHz.

To reveal the working principle of the low-pass filtering radiation response, the antenna surface current distribution at the two radiation nulls of 1.78 GHz and 1.92 GHz is shown in Figure 5. It is observed in Figure 5a that at the $Null_{1}$ frequency 1.78 GHz, the surface currents mainly concentrate on the bent metal square ring, and the currents flow in opposite directions at the horizontal and vertical direction of the square ring. Thus, it does not effectively radiate. With regard to the radiation $Null_{2}$, the currents mainly concentrate on the folded cross-dipole arms. The currents on the pair of dipole arms are oppositely distributed and thus canceled out at boresight direction.

**Figure 6:**
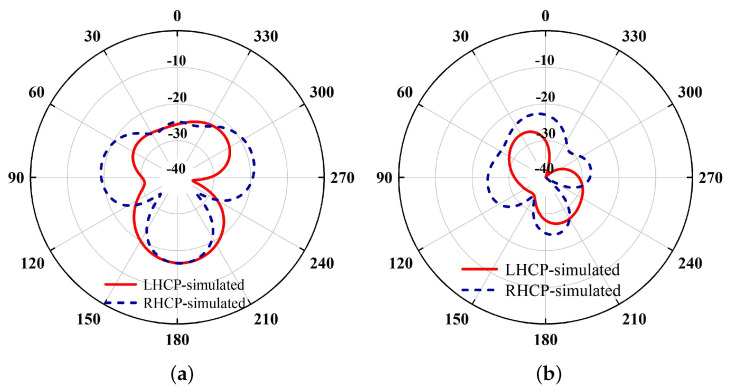
Simulated radiation patterns at the two null frequencies (**a**) 1.78 GHz and (**b**) 1.92 GHz.

The radiation patterns at these two null frequencies are illustrated in Figure 6. As seen, both the RHCP and LHCP levels are lower than −22 dBic, indicating satisfactory radiation suppression at these two frequencies. It is worth mentioning that since the two radiation nulls are respectively introduced by the bent metal square ring and the folded cross-dipole arms, their frequencies can be individually controlled by tuning the corresponding length parameters.

## 3. Antenna Implementation

**Figure 7:**
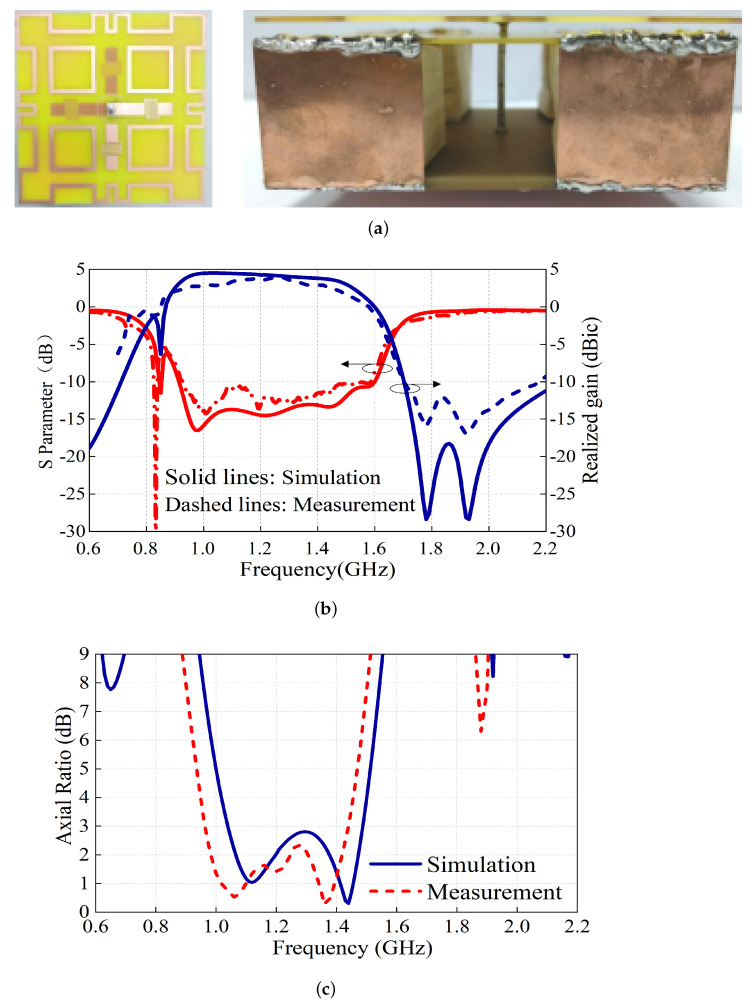
The proposed cross-dipole antenna (**a**) prototype, (**b**) measured reflection coefficients and realized gains, and (**c**) measured ARs.

Based on the above design method, the proposed compact wideband CP cross-dipole antenna with a low-pass filtering radiation response was designed, fabricated and measured, as shown in Figure 7a. The optimization was performed using a high-frequency structural simulator (HFSS) and the measurement was accomplished using an Agilent N5227A network analyzer and Satimo system. The gain of the antenna was determined using the comparative method, which involves the use of a network analyzer and a standard horn antenna as a reference for the gain. Additionally, the radiation performance was assessed by mounting the antenna on a turntable and utilizing the network analyzer to capture radiation signals from various angles. The measured results, including the S-parameters, realized gain and AR, are shown in Figure 7b,c.

**Figure 8:**
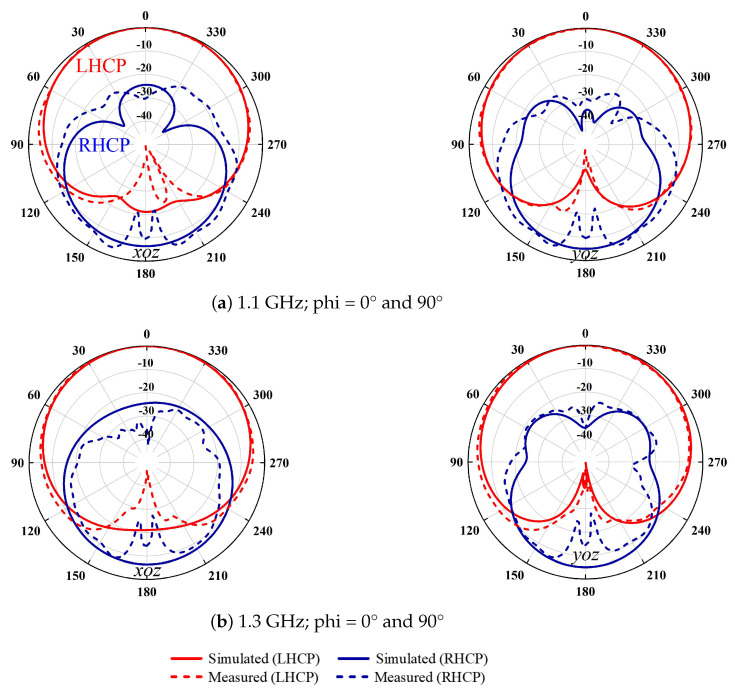
Radiation patterns of the proposed antenna at (**a**) 1.1 GHz and (**b**) 1.3 GHz (solid lines: simulation, dashed lines: measurement).

The simulation results agree well with the measurement ones. As seen in Figure 7b, stable antenna LHCP-realized gains of about 4 dBic are obtained within the operating bandwidth. The −10 dB impedance bandwidths reach 53.3% (0.92–1.59 GHz). Two radiation nulls at about 1.78 GHz and 1.92 GHz are observed as expected, ensuring a high upper-band radiation rejection level. The measured AR bandwidth is about 41% (0.95–1.44 GHz). The antenna radiation patterns at 1.1 GHz and 1.3 GHz are plotted in Figure 8. As expected, normal stable radiation patterns with low cross-polarization levels are observed. It should be mentioned that the 3 dB beamwidths (HPBW) at these two frequencies are more than 120° and, therefore, the boresight antenna-realized gains are about 3.5–4 dBic.

To address the advantages of the proposed work, the results of a comparison with other related designs are tabulated in Table 1. The filtering CP dipole antenna designs have been rarely proposed. In [5,6,7,8,9,10,17,19,20,22,24], the antennas have wide axial-ratio bandwidths but occupy large antenna sizes or heights. For example, the antenna’s axial-ratio bandwidth exceeds 90% [6,9,10], yet its dimensions are substantially large, nearly double those of the proposed antenna. In [16], folded cross-dipole arms were used to realize a compact CP antenna, but the axial-ratio bandwidth was limited to only 11.7%. In ref. [20], a shorted circuit radiator patch was used to realize a compact horizontal size CP antenna; however, the profile height surpasses 0.23 $\lambda_{0}$, and the radiation pattern is unstable. Compared with them, the proposed work simultaneously obtained a compact antenna structure $0.37\lambda_{0}\times 0.37\lambda_{0}\times 0.14\lambda_{0}$, relatively wide axial-ratio bandwidth of 41%, filtering radiation response and stable in-band realized gain. In addition, the HPBW of the proposed antenna is stable at about 120°. The wide HPBW and compact size make the proposed CP antenna very attractive in beam-scanning MIMO array antenna applications.

## 4. Conclusions

In this letter, a compact wideband circularly polarized filtering dipole antenna with a low-pass filtering radiation response has been proposed. The antenna has a compact size due to the folded cross-dipole arms and shorted circuit patches. The bent square ring and embedded four small square rings coupled to the folded cross-dipole were used to obtain wideband CP performance. A low-pass-type filtering radiation response has been obtained by the bent square ring and the folded cross-dipole structure to generate two specific radiation nulls at the upper band edges, without an extra filter circuit. As a result, two controllable radiation nulls are generated at 1.78 GHz and 1.92 GHz, respectively, as expected.

## Figures and Tables

**Table 1:** Comparison of the proposed and reported CP cross-dipole antennas.

	Size ($\lambda_{0}\times \lambda_{0}$)	Height ($\lambda_{0}$)	Imp./AR BW (%)	Gain (dBic)	Rad. Null	HPBW
[5]	0.5 × 0.5	0.25	60.5/31	6.1–6.9	No	68°
[6]	1.1 × 1.1	0.4	93.1/90.9	−2–8.6	No	N.A
[7]	0.6 × 0.6	0.21	98.2/85.5	4.5–10	No	N.A
[8]	0.6 × 0.6	0.0027	55.26/53.5	1.8–2.08	No	N.A
[9]	1.0 × 1.0	0.26	95.5/94.4	0–7	No	N.A
[10]	1.1 × 1.1	0.25	57/33	7.8–8.7	No	65°
[16]	0.2 × 0.3	0.0055	16.4/11.7	0.5–1.9	No	116°
[17]	0.5 × 0.5	0.3	40/7.3	5–6.3	No	104°
[18]	0.3 × 0.3	0.25	35.5/10.7	2.5–4.6	No	N.A
[19]	1.7 × 1.7	0.012	14.4/2.8	1.2–1.4	No	N.A
[20]	0.3 × 0.3	0.23	53.5/45	7.6–8.4	No	71°
[21]	0.4 × 0.4	0.15	92.6/71.8	−2.5–3.5	No	120°
[22]	2.4 × 0.7	0.16	34.6/23.1	5–8.8	No	N.A
[24]	0.6 × 0.6	0.16	40/19.3 49.5/33.8	4–6 4–8	No	N.A
**This work**	0.37 × 0.37	0.14	**53.3/41**	**3.5–4**	**Yes**	**120°**

## Data Availability

Data are contained within the article.

## Abbreviations

The following abbreviations are used in this manuscript:

CP	Circularly Polarized Antenna
AR	Axial Ratio
HPBW	Half-Power Beamwidth
RHCP	Right-Hand Circularly Polarized
LHCP	Left-Hand Circularly Polarized
MIMO	Multi-Input Multi-Output
